# Validating the Hypoglycaemic and Hypotensive Roles of *Salvia serotina* (Chicken Weed) in Normal Healthy Sprague–Dawley Rats

**DOI:** 10.1155/2022/6547734

**Published:** 2022-06-29

**Authors:** Tameika Bartley, Paul Reese, Sophie Turfus, Ruby Alexander-Lindo

**Affiliations:** ^1^Department of Basic Medical Sciences, Faculty of Medical Sciences, The University of the West Indies, Kingston 7, Jamaica; ^2^Department of Chemistry, Faculty of Science & Technology, The University of the West Indies, Kingston 7, Jamaica; ^3^University of Greenwich, London, UK

## Abstract

Diabetes mellitus (DM) is an endocrine disease and is characterized by hyperglycaemia. *Salvia serotina* L. (chicken weed) has been used in traditional medicine to treat various ailments including DM. Aqueous, hexane, ethyl acetate, and methanol crude extracts of *S. serotina* L. were investigated for their anti-oxidant activities and hypoglycaemic and hypotensive effects in normal, healthy Sprague–Dawley rats using the 2,2-diphenyl-1-picrylhydrazyl (DPPH) assay, oral glucose tolerance test (OGTT), and the CODA noninvasive blood pressure system to determine systolic blood pressure (SBP), diastolic blood pressure (DBP), mean arterial pressure (MAP), and heart rate (HR). The aqueous extract caused a free radical scavenging effect with an IC_50_ value of 10.2 ± 1.01 *µ*g/mL versus vitamin C (9.42 ± 1.01 *µ*g/mL). The extract lowered the blood glucose concentration at the 150 minute interval (5.00 ± 0.22 mM vs. 6.51 ± 0.33 mM; *p* = 0.004) and the 180 minute interval (4.77 ± 0.27 mM vs. 5.93 ± 0.0.30 mM; *p* = 0.015). The hexane extract gave significant hypoglycaemic activity at the 120 minute interval (4.54 ± 0.21 mmol/L vs. 5.50 ± 0.17 mmol/L; *p* = 0.005). The hexane extract also significantly lowered the SBP (132 ± 6 mm Hg; *p* = 0.014), DBP (106 ± 7 mm Hg; *p* = 0.034), and MAP (114 ± 7 mm Hg; *p* = 0.023) versus the controls SBP (156 ± 4 mm Hg), DBP (132 ± 8 mm Hg), and MAP (140 ± 6 mm Hg). Bioassay-directed purification of the hexane extract yielded 3,7,11-trimethyl-1,6,10-dodecatrien-3-ol (1), 3,7,11-trimethyl-2,6,10-dodecatrien-1-ol (2), and 5,22-stigmastadien-3*β*-ol (3) as active principles. Hence, *S. serotina* L. showed anti-oxidant, hypoglycaemic, and hypotensive effects in the rats and may have potential applications in the treatment of diabetes.

## 1. Introduction

The World Health Organization (WHO) global report on diabetes showed that the number of people living with diabetes mellitus has almost quadrupled to 422 million adults since 1980.

The disease is characterized by hyperglycaemia and causes alterations in protein, lipid, and fat metabolisms. The three major types of diabetes are type 1, type 2, and gestational. Type 2 diabetes is lifestyle-related and accounts for about 90% of all cases [1]. Effective management of diabetes mellitus includes blood glucose monitoring, diet planning, medication regime, and control of blood pressure to reduce cardiovascular risks [2].

Reactive oxygen species (ROS) are formed by the incomplete reduction of oxygen during aerobic metabolism [3], and free radicals can arise from endogenous metabolic processes and inflammation [4]. In diabetes mellitus, the free radicals produced during inflammation can react with biomolecules such as nucleic acids, proteins, lipids, and carbohydrates causing damage and progression of the disease. The elevated blood glucose concentration causes more powerful oxidizing molecules to be formed when plasma proteins become glycated [5]. Anti-oxidants can protect the body from damage by reacting with harmful free radicals. Plant-based anti-oxidants are responsible for the ability of foods to combat chronic diseases such as coronary heart disease [6].

The main pharmacological agents used to treat type 2 diabetes are the sulfonylureas, such as glibenclamide, which promote insulin secretion, and biguanides, such as metformin, which discourage the output of glucose from the liver during fasting. Other agents such as the alpha-glucosidase inhibitors are useful to diabetics in the reduction of postprandial hyperglycaemia [7]. Due to the high cost of these drugs and their unpleasant side effects, there is always a need for new therapies. One option is the use of natural products from medicinal plants. One example is the triterpenoid stigmasterol that increases serum insulin levels in type 2 diabetic rats [8]. The hypoglycaemic activities of stigmast-4-en-3-one and the corresponding alcohol, stigmast-4-en-3-ol from the bark of Anacardium occidentale (Cashew) have been reported [9]. Plants from the *Salvia* genus have been investigated for their anti-cancer, anti-microbial, and hypotensive effects. *Salvia serotina* L. is commonly called chicken weed in Jamaican and is known for its blood purifying effects [10] and is also used in conjunction with glyburide for improved diabetes management [11].

In this study, we surmise that *S. serotina* L. may have potential application in the treatment of diabetes mellitus by imparting hypoglycaemic and hypotensive effects in rats. We sought to validate the use of this plant in folklore medicine as a beneficial agent in reducing glycaemia and question whether its hypoglycaemic effect is comparable to the known oral hypoglycaemic agents: metformin and glibenclamide. Therefore, the main objectives were to investigate the anti-oxidant activity of *S. serotina* L., observe its hypoglycaemic and hypotensive effects in Sprague–Dawley rats, and, lastly, elucidate the active principles.

## 2. Materials and Methods

### 2.1. Chemicals and Reagents

Solvents (HPLC or ACS grade) were obtained from Pharmco Products, USA. Silica gel (200–425 mesh, 35–75 *μ*m dia. Type GF), dimethyl sulfoxide (DMSO), vitamin C, and 2,2-diphenyl-1-picrylhydrazyl were obtained from Sigma Chemical Co., USA. Thin-layer chromatography plates (0.2 mm, UV fluorescent indicator) were obtained from Macherey-Nagel, GmbH Co. KG Germany. Glucose and corn oil were obtained from the local supermarket.

### 2.2. Equipment

Büchi rotary evaporator (R-124 Switzerland) was used for rotary evaporation. Bruker Avance NMR Spectrometers (500 MHz and 200 MHz) were used for ^1^H-NMR and ^13^C-NMR spectroscopy. FTIR spectroscopy was done using a Bruker Vector 22 IR spectrometer. The GC-MS was carried out by Agilent Technologies gas chromatograph connected to a mass spectrometric detector Agilent MSD 5973N. Melting point analysis was done using a standard capillary tube and melting point machine. UNICO 1100RS spectrophotometer was used for the anti-oxidant assay; Accu-Chek Active glucometer and Accu-Chek Active glucose test strips were obtained from Lasco Pharmaceuticals Co. Ja. Ltd. CODA noninvasive blood pressure machine was obtained from Kent Scientific, USA.

### 2.3. Experimental Procedures

#### 2.3.1. Plant Material

*S. serotin*a L. (chicken weed) was collected in St. Elizabeth, Jamaica. The plant was authenticated at the Herbarium, Department of Life Sciences, UWI, Mona, and a voucher specimen was deposited with UWI (accession number, 36358).

#### 2.3.2. Plant Preparation and Extraction

The aerial sections of *S. serotina* L. (chicken weed) were air-dried at room temperature. The plant was then milled to yield a finely ground material, which was weighed and packed into a glass column. The plant was then extracted sequentially using hexane, ethyl acetate, and methanol. The extracts were filtered, and the solvent was then evaporated in vacuo to yield hexane, ethyl acetate, and methanol crude extracts. The aqueous extract was prepared by heating the milled plant (40 g) in distilled water (400 mL) while gently stirring on a hot plate for 12 hours with intermittent heating at 45°C. The mixture was filtered through a sieve, and the filtrate was evaporated by gentle heating at 60°C to yield a dark brown viscous liquid. Further evaporation was allowed at room temperature until a brittle material resulted. These four extracts were kept refrigerated for biological assays, purification, and elucidation of active natural compounds.

#### 2.3.3. Phytochemical Screening

Phytochemical examinations were performed on the three crude extracts using standard methods described in the literature with minor modifications [12, 13].

#### 2.3.4. Anti-Oxidant Assay

The method used was similar to that described by Doss and Pugalenthi in 2012 with minor modifications [14]. DPPH is a stable radical with oxidizing power that forms a purple solution with methanol. DPPH becomes reduced when it reacts with an anti-oxidant whereby it accepts a proton and the solution changes from purple to yellow [15].

The plant extracts or ascorbic acid were weighed (4 mg) and dissolved in methanol (4 mL) to make up a stock concentration of 1 mg/mL. The stock solutions were diluted serially to yield working concentrations of 200, 100, 50, 25, 10, and 1 *µ*g/mL. 2,2-Diphenyl-1-Picrylhydrazyl (DPPH) was weighed (9.86 mg) and added to methanol (250 mL) to make a standard solution at 0.1 mM.

Two milliliter of each stock solution at each concentration was pipetted into a clean glass tube, and 2 mL of the DPPH standard solution was added to the tube. Negative control was set up by combining methanol (2 mL) and DPPH solution (2 mL). The tubes were incubated in the dark at room temperature for 45 minutes. Absorbance was taken at 517 nm using a UV-Vis spectrophotometer.

A radical scavenging activity was expressed as a 1% scavenging activity and calculated using the following formula:

(1) $$\begin{array}{c} \text{Radical Scavenging Activity},I_{\text{DPPH}}\left(\%\right)=\left(\frac{\text{OD Control}-\text{OD Sample}}{\text{OD Control}}\right)\times 100. \end{array}$$

The *I*_DPPH_ % was plotted against the sample concentration, and a logarithmic regression curve was established in order to calculate the IC_50_ value (inhibitory concentration), which represents the concentration of the sample (*µ*g/mL), which is needed to decrease the absorbance of DPPH by 50%.

#### 2.3.5. Biological Study

Approval for the use of Sprague–Dawley (S–D) rats in all experiments was obtained from the UWI (Mona) Ethics Committee (AN 20, 13/14). These animals were housed in stainless steel cages and acclimatized at the Animal House in the Department of Basic Medical Sciences (BMS) at the University of the West Indies, Mona. They were maintained there under the supervision of the animal care staff and a veterinarian consultant. All animals were housed at room temperature under 12-hour cycles of dark and light and were fed tap water and Purina Laboratory Diet ad libitum. Each experimental group had 6 rats to allow for statistical analysis.

#### 2.3.6. Oral Glucose Tolerance Test (OGTT)

The OGTT was performed using the methods described by Wilson and Islam in 2012 and Rao and Naidu in 2010 with minor modifications [16, 17]. Normal, healthy S–D rats (250–400 g) fasted overnight for approximately 12 hours. A fasting blood glucose reading was taken by making an incision at the tip of the tail, and a drop of blood was placed onto the Accu-Chek Active glucose test strip that was inserted into the associated glucometer. The crude extracts (0.3 mL) were then administered orally at 200 mg/kg body weight (BW) or intravenously (IV) at 50 mg/kg BW. The five semipurified fractions TBHeFR1 to TBHeFR5 were administered intravenously at 30 mg/kg BW. Recrystallization of TBHeFR5 yielded TBHeFR5II, which was administered intravenously at 10 mg/kg·BW. Control rats were treated with 0.3 mL of dimethyl sulfoxide (DMSO) for IV administration or cooking oil for oral administration. Blood glucose readings were then taken at the 30 and 60 minute intervals. Immediately following, a glucose load (1.75 g/kg BW; 0.3 mL) was administered orally in warm distilled water. Blood glucose readings were taken at 30 minute intervals for 2½ hours.

#### 2.3.7. Blood Pressure Test

Haemodynamic parameters were determined using the CODA noninvasive blood pressure test and the CODA machine from Kent Scientific Corporation, USA [18]. Rats were trained by subjecting them to the experimental conditions prior to the actual blood pressure determination. Four parameters were observed: systolic blood pressure (SBP), diastolic blood pressure (DBP), mean arterial pressure (MAP), and heart rate (HR). The occlusion pressure recording cuff (O-cuff) was placed around the tail of the animal. This was followed by the volume pressure recording cuff (V-cuff). Basal readings were taken for 5 minutes, and the cuffs were removed. The extract (0.3 mL) was then administered intravenously at 50 mg/kg BW. Control rats were treated with 0.3 mL of dimethyl sulfoxide (DMSO). Immediately following, the O-cuff and V-cuff were replaced, and SBP, DBP, MAP, and HR were recorded for 20 minutes.

#### 2.3.8. Purification of the Hypoglycaemic Crude Extract

The hexane extract of chicken weed indicated hypoglycaemic activity. Chromatography separations were carried out using standard techniques [19, 20]. The hexane extract (5.13 g) was fractionated by column chromatography on silica gel (200–425 mesh, stationary phase height of 30 cm) usinggradient elution with hexane and dichloromethane as the mobile phase. Fractions were collected in 50 mL portions and analyzed on thin-layer chromatography (silica gel) using a solvent system of hexane:dichloromethane (2:3). A total of 43 fractions were obtained, and fractions with similar TLC (silica gel) profiles were grouped resulting in 5 main fractions (TBHeFR1 to TBHeFR5), and the solvent was removed under reduced pressure at 50°C using a rotary evaporator. The fraction TBHeFR1 (1.852 g) was an intense red-orange solid, TBHeFR2 (0.1568 g) an orange-coloured solid material, TBHeFR3 (1.057 g) a red-orange gel, TBHeFR4 (0.5272 g) a yellow-orange solid, and TBHeFR5 (1.794 g) a dark-green solid material with yields of 36%, 0.03%, 0.21%, 0.10%, and 35%, respectively. These major fractions were bioassayed using the OGTT on normoglycaemic Sprague–Dawley rats in order to carry out a bioassay-guided isolation of the active principles. Both TBHeFR3 and TBHeFR5 revealed a lowering in blood glucose levels. TBHeFR5 formed colourless needles on standing and was recrystallized using cold hexane. A white crystalline substance (15.5 mg) was obtained and detected at 254 nm as a blue fluorescent band with an *R*_*f*_ value of 0.1 when chromatographed with 7:1 hexane:ethyl acetate. TBHeFR3 gave a yellow-orange oil (TBHeFR3C, 0.4817 g) when chromatographed in an ethyl acetate:hexane (2:3) solvent system.

#### 2.3.9. Structure Elucidation

Structural elucidation was carried out using gas chromatography-mass spectrometry (GC-MS), ^1^H-NMR, ^13^C-NMR, FTIR spectroscopy, as well as melting point analysis. Chemical structures were created using ACD/Chem Sketch Software version 14.

### 2.4. Statistical Analysis

Graphical data for inhibition assays were analyzed using GraphPad Prism version 7.03. Readings for the OGTT were obtained in duplicates, and means were calculated. All readings for the haemodynamic parameter tests were collected and averaged for each 5 minute interval. Values were reported as mean ± standard error of the mean. Data were analyzed using Statistics Package for the Social Sciences (SPSS) version 22 and Student's *t-*test with *p* < 0.05 considered to be significant.

## 3. Results

### 3.1. Phytochemical Screening

The active crude hexane extracts contained terpenoids, steroids, and tannins (Table 1). The more polar solvents extracted a wider range of phytochemicals.

### 3.2. Anti-Oxidant Assay

**Figure 1:**
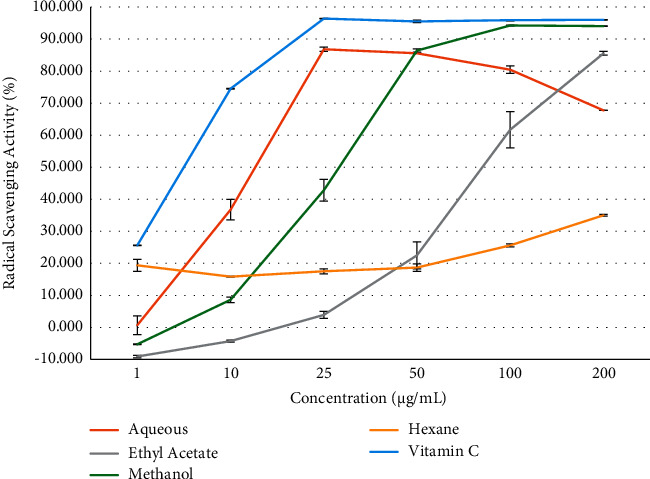
The free radical scavenging assay of the crude extracts when compared with the known anti-oxidant vitamin C.

The most potent radical quenching occurred when a concentration of 25 *µ*g/mL of the extract or vitamin C was administered (Figure 1).

The anti-oxidant activity of the aqueous extract was most similar to vitamin C. The IC_50_ value for the aqueous extract was 10.21 ± 1.01 *µ*g/mL while vitamin C had the IC_50_ value of 9.42 ± 1.01 *µ*g/mL. Both the aqueous and methanol extracts were expected to display an anti-oxidant effect since they contain phytochemicals such as flavonoids, as reported in Table 1, which are popularly known for their anti-oxidative properties [21]. The methanol extract gave an IC_50_ value of 24.22 ± 1.05 *µ*g/mL when compared with vitamin C. The methanol and aqueous extracts contained the same set of phytochemicals (Table 1). However, the methanol extract required a higher concentration than the aqueous extract in order to impart a radical scavenging activity that was similar to vitamin C. The less polar ethyl acetate and hexane extracts showed the lowest radical scavenging potential with IC_50_ values of 63.28 ± 1.11 *µ*g/mL and 94.00 ± 1.02 *µ*g/mL, respectively.

### 3.3. Biological Activities

#### 3.3.1. Oral Glucose Tolerance Test (OGTT)

**Figure 2:**
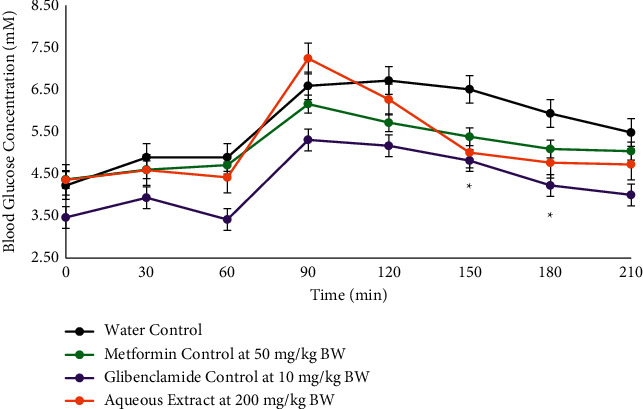
OGTT of the aqueous crude extract from *Salvia serotina L.* when administered orally at 200 mg/kg BW versus water control and oral hypoglycaemic agents glibenclamide and metformin positive controls. *n* = 6 rats. ^*∗*^ − *p*=0.004 at 150 min and ^*∗*^ − *p*=0.015 at 180 min.

The aqueous extract caused a significant decrease in the blood glucose concentration when compared to metformin and glibenclamide (Figure 2) at the 150 and 180 minute intervals (5.01 ± 0.22 vs. 6.51 ± 0.33; *p* = 0.004, and 4.77 ± 0.27 vs. 5.93 ± 0.30; *p* = 0.015), respectively.

**Figure 3:**
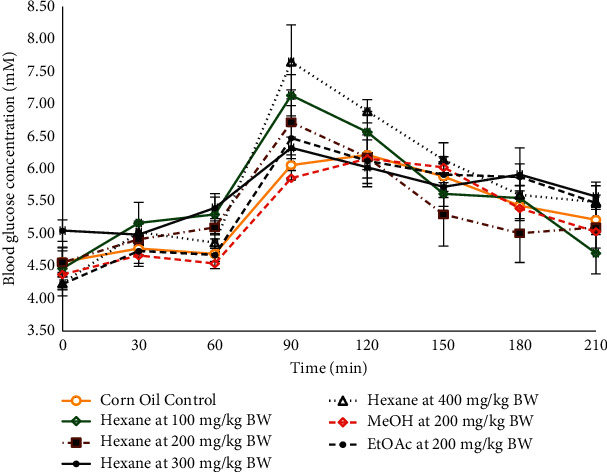
The OGTT of the crude extracts when administered orally versus corn oil control (*n* = 6). EtOAc – ethyl acetate and MeOH – methanol.

The ethyl acetate and methanol crude extracts imparted an effect similar to the control, but the hexane extract indicated a drastic decrease in the blood glucose from the 90 min interval at 6.72 ± 0.16 mM to 5.01 ± 0.28 mM at the 180 min interval (Figure 3). Hence, an oral dose-dependency test was carried out using the hexane extract at concentrations of (100, 200, 300, and 400) mg/kg BW.

**Figure 4:**
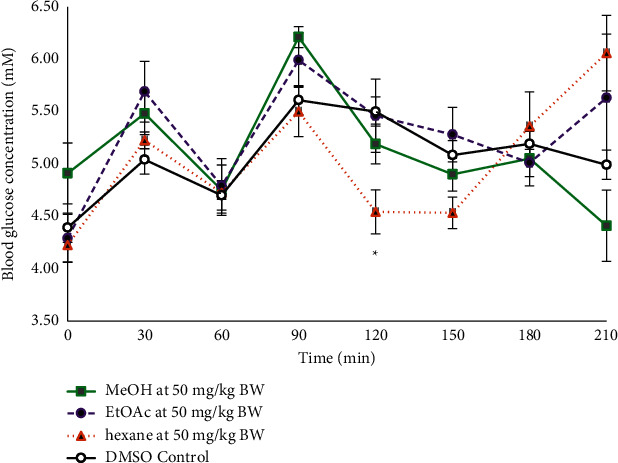
OGTT of crude extracts from *Salvia serotina L.* (chicken weed) when administered intravenously at 50 mg/kg BW versus DMSO control (*n* = 6). ^*∗*^ − *p* < 0.05. EtOAc – ethyl acetate and MeOH – methanol.

The dose-dependency test showed that the hexane crude extract was most effective when administered at 200 mg/kg BW. An intravenous administration was also performed (Figure 4) where the crude hexane extract indicated a significant lowering of the blood glucose concentration at the 120 to 150 min time intervals.

**Figure 5:**
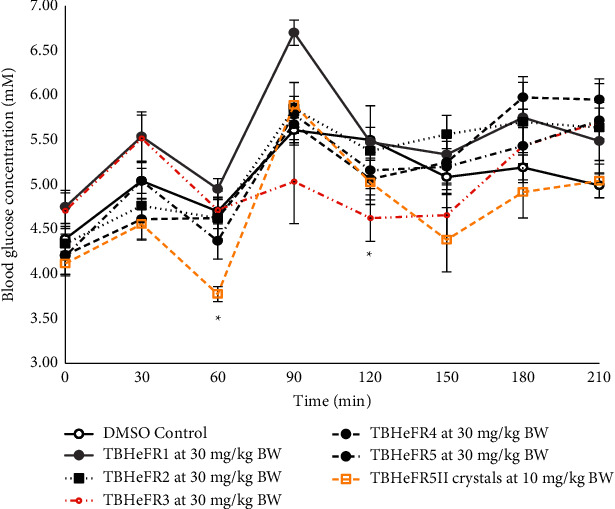
OGTT of semipurified fractions of hexane crude extract when administered intravenously versus DMSO control (*n* = 6). ^*∗*^ − *p* < 0.05.

Purification of the active hexane extract was performed using chromatography techniques. Based on the OGTT of the semipurified fractions, the most effective fraction was TBHeFR3 that showed hypoglycaemic activity throughout the postprandial region (Figure 5). The most significant hypoglycaemic activity was observed at the 120 min interval when compared with the control (4.63 ± 0.26 mM vs. 5.50 ± 0.17 mM).

TBHeFR5 was recrystallized to give TBHeFR5II that was administered intravenously (Figure 5). The results showed that as the sample was purified, the hypoglycaemic effect became more significant. There was a decrease in the blood glucose concentration in both the fasting and postprandial regions, significantly at the 60 minute interval when compared with the DMSO control (3.78 ± 0.14 mM vs. 4.70 ± 0.25 mM; *p*=0.01). Thus, indicating that TBHeFR5II (stigmasterol) was responsible for the hypoglycaemic effect observed.

#### 3.3.2. Blood Pressure

**Figure 6:**
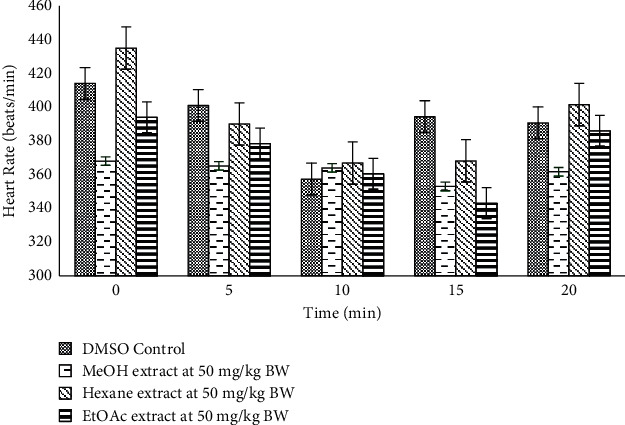
The effects of crude extracts from *S. serotina* on heart rate versus DMSO control when administered intravenously at 50 mg/kg BW (*n* = 6).

The hexane crude extract displayed the most effective hypotensive effect in the rats causing a lowering of the SBP (*p*=0.01 and *p* = 0.02), DBP (*p*=0.03 and *p*=0.002), and MAP (*p*=0.02 and *p*=0.002) at 10 and 15 minutes, respectively, postextract administration (Table 2). However, there was no effect on heart rate (Figure 6).

### 3.4. Gas Chromatography-Mass Spectrometry (GC-MS)

**Figure 7:**
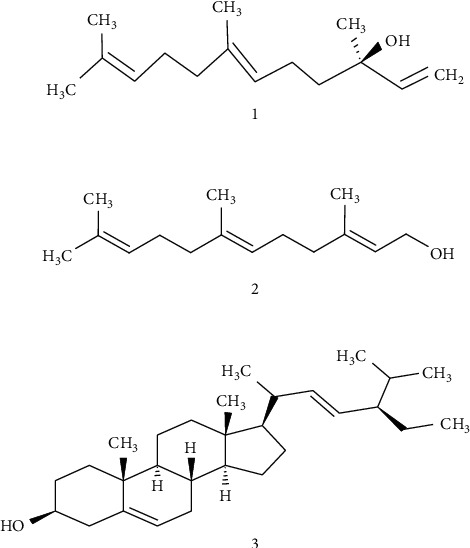
The active compounds nerolidol (1), farnesol (2), and stigmasterol (3) were isolated from the crude hexane extract of *Salvia serotina* L.

The GC-MS analysis of the active crude hexane fraction TBHeFR3C presented two compounds of interest: 3,7,11-Trimethyl-1,6,10-dodecatrien-3-ol commonly called Nerolidol and 3,7,11-Trimethyl-2,6,10-dodecatrien-1-ol commonly called Farnesol (Figure 7) based on the GC-MS spectra (Figure S1) and the National Institute of Standards and Technology (NIST) database.

The fraction, TBHeFR5, was charred when sprayed with sulfuric acid (10%) after heating. This fraction yielded a white crystalline solid after recrystallization, TBHeFR5II, which gave a purple colour with ammonium molybdate and sulfuric acid spray, indicating the presence of a sterol. Detailed ^1^H-NMR, ^13^C-NMR, and IR spectral analysis, with a melting point value of 134°C that coincides with the value from the literature of 135°C [22], indicated the presence of the steroid 5,22-stigmastadien-3*β*-ol (**2**), commonly called stigmasterol (Figure 7).

### 3.5. Spectral Analysis for Stigmasterol

The ^1^H-NMR spectra (Figure S2), ^13^C-NMR (Figure S3), and infrared spectra (Figure S4) were analyzed as follows: ^**1**^**H-NMR** (CDCl_3_, 500 MHz) *δ*: 1.35 (2H, m), 1.83 (2H, m), 3.55 (1H, m), 7.25 (1H, s), 1.65 (2H, m), 5.34 (1H, m), 5.45 (1H, d), 1.97 (2H, m), 1.95 (2H, d), 0.94 (1H, m), 1.05 (3H, s), 2.04 (2H, d), 2.00 (2H, m), 0.65 (3H, s), 1.01 (1H, m), 1.56 (2H, m), 2.25 (2H, m), 1.15 (1H, q), 0.70 (3H, s), 1.01 (3H, s), 0.95 (1H, d), 1.03 (3H, d), 5.05 (1H, s), 5.2 (1H, s), 0.85 (1H, d), 1.55 (1H, m), 0.85 (3H, d), 0.80 (3H, d), 1.43 (2H, m), and 0.81 (3H, t; Table S1).

^**13**^ **C-NMR** (CDCl_3_, 500 MHz) *δ*: 39.69, 31.66, 71.83, 42.29, 140.76, 121.75, 31.90, 37.26, 50.13, 40.52, 21.23, 45.83, 24.32, 28.94, 55.95, 11.83, 19.42, 56.77, 21.09, 138.34, 129.27, 51.25, 18.76, 20.64, 19.42, and 25.43 for C15-28 (Table S1).

**FTIR** (Quartz) cm^−1^: 837, 961, 1,056, 1,373, 1,457, 1,538, 1,702, 2,859, 2,937, and 3,460 (Table S2). These spectral results corroborate with previously obtained data for stigmasterol [23–25].

## 4. Discussion

### 4.1. Phytochemical Analysis of *Salvia serotina L*. Extracts

The main objectives of the study were to investigate the effects of *S. serotina* L. (chicken weed) on the blood glucose concentration and blood pressure in normal Sprague–Dawley rats, observe the anti-oxidant potency of the crude extracts, and identify the active hypoglycaemic principles. *S. serotina* contained glycosides, saponins, flavonoids, terpenoids, tannins, and steroids based on the phytochemical analysis (Table 1). It is the presence of such phytochemicals in herbs, fruits, and vegetables that is responsible for the health benefits provided by these plants [26, 27], and so the active principles can serve as potential therapies in diabetes.

### 4.2. Anti-Oxidant Activities of *Salvia serotina* L. Extracts

The hyperglycaemia that precedes frank diabetes mellitus causes oxidative stress [28] due to the formation of advanced glycated end products (AGEs). These AGEs lead to the formation of reactive oxygen species (ROS) that are free radicals [29]. It was necessary to study the anti-oxidant effect of the various crude extracts as the phytochemical screening of the crude extracts (Table 1) indicated the presence of phenolics such as flavonoids, which have known anti-oxidative effects. The aqueous extract showed a free-radical scavenging effect that was similar to the known anti-oxidant, vitamin C (Figure 2). The most potent radical quenching effect occurred when a concentration of 25 *µ*g/mL of vitamin C was administered. At this concentration, the IC_50_ value for the aqueous extract was 10.21 ± 1.01 *µ*g/mL, while vitamin C had the IC_50_ value of 9.42 ± 1.01 *µ*g/mL. Both the aqueous and methanol extracts contained phytochemicals such as flavonoids (reported in Table 1), which are popularly known for their anti-oxidative properties. The difference in anti-oxidative property may be due to a greater concentration of the radical scavenging components being extracted in the more polar solvent, water, when compared with methanol. Anti-oxidant activity is dependent on the amount of total phenolic compounds, and so it can be ascertained that the aqueous extract had a larger variety of polyphenols than the methanol extract, which had an IC_50_ value of 24.22 ± 1.05 *µ*g/mL. The ethyl acetate and hexane extracts showed weaker anti-oxidant effects because of their nonpolar properties, and so less of the radical scavenging compounds were removed in these extracts. Compounds that antagonize the effects of reactive oxygen species are effective in ameliorating oxidative stress and in the lowering blood pressure [30], and so this plant may have applications in the treatment of diabetic complications.

### 4.3. Hypoglycaemic Effects of *Salvia serotina* L. Extracts

The hexane, ethyl acetate, methanol, and aqueous crude extracts were bioassayed by oral administration in Sprague–Dawley (S–D) rats at 200 mg/kg body weight (BW). The hexane crude extract caused a significant decrease in the blood glucose concentration at the 120 minute interval, and the aqueous crude extract gave a similar effect as metformin at the 150 and 180 minute intervals (Figure 1). The 120 minute interval is crucial in oral glucose tolerance tests, and so the hexane crude extract was used to carry out an oral dose-dependency test at concentrations of 100, 200, 300, and 400 mg/kg BW. The dosage dependency study highlighted 200 mg/kg BW as the most effective dosage. At higher levels of extract, the assimilation of harmful phytochemicals may cause an impairment in the insulin response and cause an increase in the blood glucose concentration as seen in Figure 3 since the excess amounts of polyphenols from plant species can be harmful [31]. Based on the results from the oral administration of the plant extracts, an intravenous administration of the plant extract at 50 mg/kg BW was carried out using DMSO as the carrier (Figure 4). There was a significant lowering of the blood glucose concentration in the postprandial region at the 120 minute (4.54 ± 0.21 mM; *p*=0.005) time interval when compared with the control (5.50 ± 0.17 mM). The intravenous mode of administration of the extract enabled the direct assimilation of the phytochemical into the bloodstream and retarded the possible degradation by enzymes in the gastrointestinal tract. It was also observable that the blood glucose concentration increased again after the 120 minute decline (Figure 4) and so is also possible that sufficient amounts of the naturally isolated hypoglycaemic components did not enter the blood to cause any long-term effect during the three hours of the OGTT. When the extracts are in their crude states, there is a diversity of compounds present including glycosides that could contribute to a rise in the glycaemic peak. Additionally, the phytochemical compounds present could be only moderately absorbed [32] or slow-acting and may be more effective if the rats were treated continuously over a period of time to cause an accumulation in the blood. The semipurification of the hexane extract revealed TBHeFR3 that caused a lowering of the glycaemic peak and significantly lowered the blood glucose concentration at the 120 minute interval (Figure 5), showing a more improved blood glucose lowering effect than the crude hexane extract.

### 4.4. Hypotensive Effects of *Salvia serotina* L. Extracts

Since hypertension is a major complication of diabetes mellitus, it was plausible to investigate the effect that a hypoglycaemic agent imposes on blood pressure. *Salvia* species have been used in ethnomedicine to reduce blood pressure by the inhibition of angiotensin-converting enzyme (ACE) [32]. Hypertension poses a great risk for cerebrovascular disease and coronary heart disease [33]. The CODA noninvasive blood pressure machine (Kent Scientific) was used to bioassay the hexane, ethyl acetate, and methanol crude extracts in normotensive S–D rats. The presence of saponins and flavonoids, which are marked vasodilators, was observed in the methanol and ethyl acetate extracts (Table 2), and so it was expected that these extracts would have conveyed a hypotensive effect, but this was not observed. The hexane crude extracts significantly lowered systolic blood pressure (SBP), diastolic blood pressure (DBP), and mean arterial pressure (MAP) from 10 minutes postextract administration, while there was no significant effect on heart rate (Figure 6). *Salvia* species have been popular for various bioactivities such as anti-inflammatory and hypotensive effects in the treatment of nervous conditions and circulation disorders [34]. Therefore, it is necessary to pursue further studies into the mechanism of action of *S. serotina* L. (chicken weed) in the lowering of blood pressure. In light of these observations, it was necessary to purify the hexane extract in order to identify the active compounds.

### 4.5. Bioassay Guided Purification of the Hexane Crude Extract

The purification of the hexane extract afforded the plant sterol 5,22-stigmastadien-3*β*-ol, commonly called stigmasterol (Figure 7), as one of the active hypoglycaemic principles from the plant following chromatographic and spectroscopic analyses. The intravenous administration of stigmasterol (TBHeFR5II) crystals at 10 mg/kg BW (Figure 5) caused a reduction in the blood glucose concentration during the fasting and postprandial regions when compared with the DMSO control. Stigmasterol has beneficial bioactivities such as anti-oxidant, anti-hypercholesterolemic, and hypoglycaemic effects in diabetic rats [35]. The other two compounds of interest, Farnesol and Nerolidol, were present in fraction TBHeFR3. Oral administration of Farnesol caused a lowering of the glycaemic peak and reduced glycaemia in streptozotocin-induced type 2 diabetic mice by inhibition of alpha-glucosidase enzyme [36], while Nerolidol displayed various bioactivities both *in vitro* and *in vivo* by showing its anti-ulcer, anti-oxidant, and anti-inflammatory properties [37], thereby indicating the anti-diabetic properties of these active compounds. Overall, the results from Figures 3–5 show that the naturally isolated compounds seemed to be slow-acting, while the crude hexane extract was administered possibly due to the other components that were present. Some of these compounds could be hyperglycaemic in nature, and hence, they imparted an antagonistic effect on the active hypoglycaemic principle(s). Additionally, the active compounds present may have taken a long time to be metabolized due to physicochemical properties such as lipophilicity. Overall, the results of this study validate the use of *S. serotina* L. as a blood purifier in folklore medicine due to the anti-oxidant, hypopglycaemic, and hypotensive effects that were observed.

## 5. Conclusion

The phytochemical screening of *S. serotina* L. (chicken weed) revealed the presence of glycosides, saponins, terpenoids, tannins, flavonoids, and steroids that are useful compounds with various biological activities. The crude hexane extract caused hypoglycaemic and hypotensive activities in the S–D rats. *S. serotina* L. (chicken weed) aqueous extract had hypoglycaemic and anti-oxidant effects that were comparable to metformin and vitamin C, respectively. The results indicated that *S. serotina* L. demonstrated useful bioactivities as an anti-oxidant, hypoglycaemic, and hypotensive agent. The plant provided some active fractions for hypoglycaemic agents and afforded 5,22-stigmastadien-3*β*-ol (stigmasterol), 3,7,11-trimethyl-2,6,10-dodecatrien-1-ol (Farnesol), and 3,7,11-trimethyl-1,6,10-dodecatrien-3-ol (Nerolidol) that are known as hypoglycaemic phytoconstituents. Therefore, *S. serotina* L. may have potential applications in the development of effective therapies for type 2 diabetes.

## Figures and Tables

**Table 1:** Phytochemical screening of the various crude extracts from *Salvia serotina L.* (chicken weed).

Phytochemical	Crude hexane extract	Crude ethyl acetate extract	Crude methanol extract	Crude aqueous
Glycoside	−	+	+	+
Saponin	−	+	+	+
Flavonoid	−	+	+	+
Terpenoid	+	−	+	+
Tannin	+	+	+	+
Alkaloid	−	−	−	−
Steroid	+	+	−	−

*Note.* + means present, and − means absent.

**Table 2:** The effects of crude extracts from *Salvia serotina L.* on SBP, DBP, and MAP when administered intravenously at 50 mg/kg BW in normotensive S–D rats. (*n* = 6). ^*∗*^ − *p* < 0.05.

Experimental group	Blood pressure (mm·Hg)	Time (min)				
		0	5	10	15	20
DMSO control	SBP	129 ± 4	140 ± 8	156 ± 4	156 ± 3	152 ± 4
MeOH extract		119 ± 3	120 ± 6	135 ± 11	142 ± 8	144 ± 8
EtOAc extract		122 ± 3	147 ± 7	153 ± 4	154 ± 6	161 ± 6
Hexane extract^a^		123 ± 4	130 ± 8	132 ± 4^*∗*^	129 ± 3^*∗*^	132 ± 4

DMSO control	DBP	96 ± 8	114 ± 9	132 ± 8	136 ± 4	129 ± 6
MeOH extract		92 ± 4	103 ± 7	119 ± 12	123 ± 7	129 ± 8
EtOAc extract		91 ± 5	120 ± 9	126 ± 5	131 ± 5	131 ± 4
Hexane extract^b^		83 ± 6	104 ± 8	106 ± 7^*∗*^	103 ± 6^*∗*^	104 ± 7

DMSO control	MAP	106 ± 6	123 ± 9	140 ± 6	142 ± 3	137 ± 5
MeOH extract		107 ± 6	107 ± 7	124 ± 12	129 ± 8	133 ± 8
EtOAc extract		101 ± 5	129 ± 8	135 ± 4	138 ± 6	141 ± 4
Hexane extract^c^		96 ± 4	112 ± 7	115 ± 7^*∗*^	111 ± 7^*∗*^	113 ± 7

*Note.* SBP – systolic blood pressure, DBP – diastolic blood pressure, MAP – mean arterial pressure, DMSO – dimethyl sulfoxide, MeOH – methanol, and EtOAc – ethyl acetate. ^a^The hexane extract significantly lowered the SBP at the 10 and 15 minutes intervals (*p*=0.01 and *p*=0.02, respectively). ^b^The hexane extract significantly lowered the DBP at the 10 and 15 minutes intervals *p*=0.03 and *p*=0.002, respectively. ^c^The hexane extract significantly lowered the MAP at the 10 and 15 minutes intervals (*p*=0.02 and *p*=0.002, respectively).

## Data Availability

The data sets generated and analyzed during the study are available from the corresponding author on reasonable request.

## Abbreviations

DM: Diabetes mellitus
WHO: World Health Organization
ROS: Reactive oxygen species
OGTT: Oral glucose tolerance test
SBP: Systolic blood pressure
DBP: Diastolic blood pressure
MAP: Mean arterial pressure
HR: Heart rate
DMSO: Dimethyl sulfoxide
AGE: Advanced glycated end product
DPPH: 2,2-Diphenyl-1-picrylhydrazl
HNMR: Proton nuclear magnetic resonance
CNMR: Carbon-13 nuclear magnetic resonance
FTIR: Fourier transform infrared
GC-MS: Gas chromatography-mass spectrometry

## Supplementary Materials

Supplementary Materials Figure S1. The gas chromatogram of TBHeFR3 showed two bioactive compounds of interest, namely 3,7,11-trimethyl-1,6,10-dodecatrien-3-ol (1) commonly called Nerolidol and 3,7,11-trimethyl-2,6,10-dodecatrien-1-ol (2) commonly called Farnesol. Figure S2. The ^1^H-NMR spectrum for fraction TBHeFRII that was elucidated as stigmasterol. Figure S3. The ^13^C-NMR spectrum for fraction TBHeFRII that was elucidated as stigmasterol. Figure S4. The FTIR spectrum for fraction TBHeFRII that was elucidated as stigmasterol. Table S1. The ^1^H-NMR and ^13^C-NMR spectral analysis of TBHeFRII in CDCl_3_ at 500 MHz when compared with the literature [23, 24]. Table S2. FTIR spectral data showing the functional groups detected in TBHeFR5II when compared with the literature [25].
