# Supplementary material for: Validating the Hypoglycaemic and Hypotensive Roles of Salvia serotina (Chicken Weed) in Normal Healthy Sprague–Dawley Rats
Source: ScientificWorldJournal. 2022 Jun 29;2022:6547734. doi: 10.1155/2022/6547734 (PMC9259359; doi:10.1155/2022/6547734)
Supplement: Supplementary Materials — Figure S1. The gas chromatogram of TBHeFR3 showed two bioactive compounds of interest, namely 3,7,11-trimethyl-1,6,10-dodecatrien-3-ol (1) commonly called Nerolidol and 3,7,11-trimethyl-2,6,10-dodecatrien-1-ol (2) commonly called Farnesol. Figure S2. The 1H-NMR spectrum for fraction TBHeFRII that was elucidated as stigmasterol. Figure S3. The 13C-NMR spectrum for fraction TBHeFRII that was elucidated as stigmasterol. Figure S4. The FTIR spectrum for fraction TBHeFRII that was elucidated as stigmasterol. Table S1. The 1H-NMR and 13C-NMR spectral analysis of TBHeFRII in CDCl3 at 500 MHz when compared with the literature [23, 24]. Table S2. FTIR spectral data showing the functional groups detected in TBHeFR5II when compared with the literature [25]. [file 6547734.f1.zip › 6547734.f1/FIGURE S2.pdf]

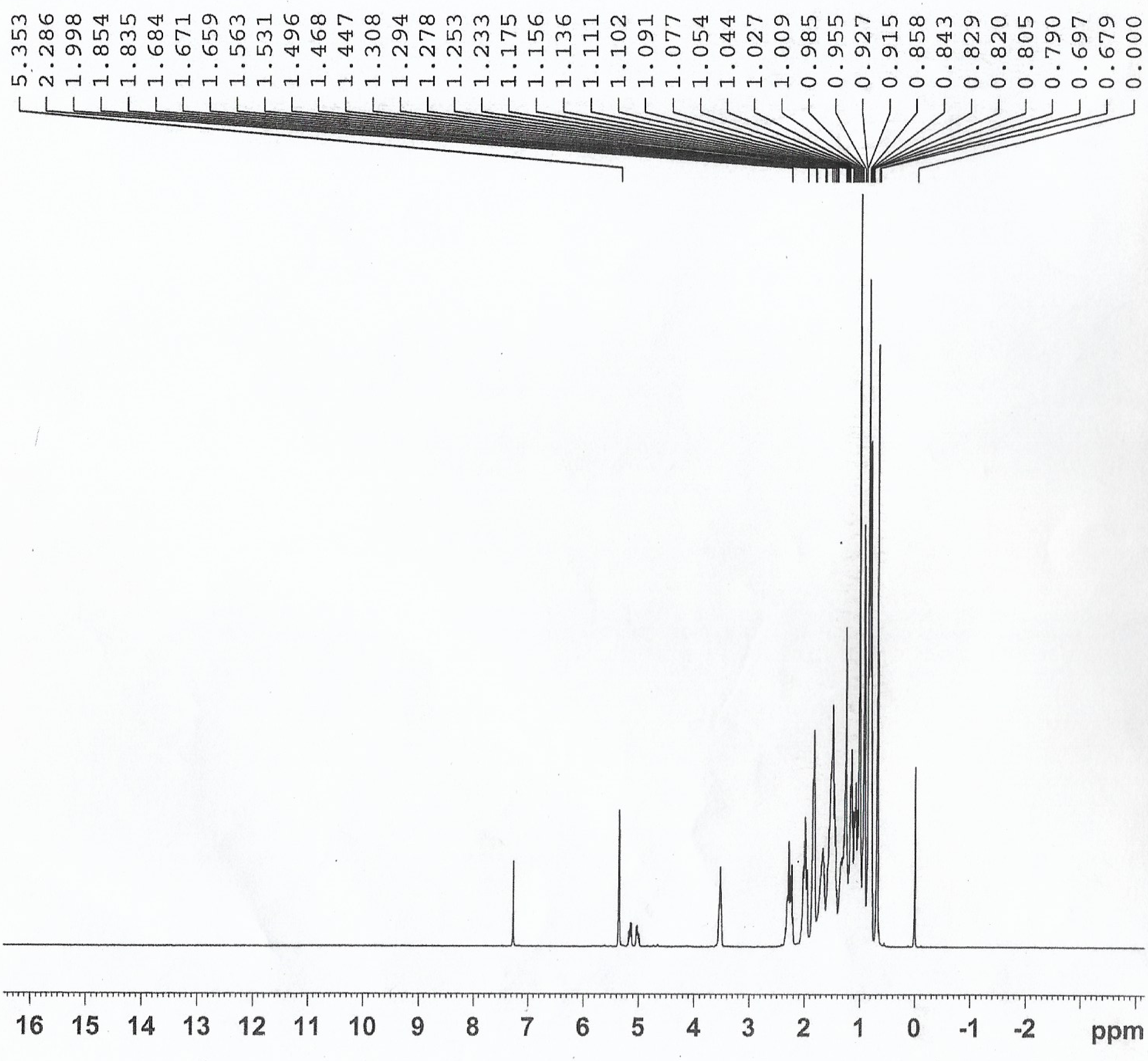
5.353
2.286
1.998
1.854
1.835
1.684
1.671
1.659
1.563
1.531
1.496
1.468
1.447
1.308
1.294
1.278
1.253
1.233
1.175
1.156
1.136
1.111
1.102
1.091
1.077
1.054
1.044
1.027
1.009
0.985
0.955
0.927
0.915
0.858
0.843
0.829
0.820
0.805
0.790
0.697
0.679
0.000
16 15 14 13 12 11 10 9 8 7 6 5 4 3 2 1 0 -1 -2
ppm
