# Supplementary material for: Validating the Hypoglycaemic and Hypotensive Roles of Salvia serotina (Chicken Weed) in Normal Healthy Sprague–Dawley Rats
Source: ScientificWorldJournal. 2022 Jun 29;2022:6547734. doi: 10.1155/2022/6547734 (PMC9259359; doi:10.1155/2022/6547734)
Supplement: Supplementary Materials — Figure S1. The gas chromatogram of TBHeFR3 showed two bioactive compounds of interest, namely 3,7,11-trimethyl-1,6,10-dodecatrien-3-ol (1) commonly called Nerolidol and 3,7,11-trimethyl-2,6,10-dodecatrien-1-ol (2) commonly called Farnesol. Figure S2. The 1H-NMR spectrum for fraction TBHeFRII that was elucidated as stigmasterol. Figure S3. The 13C-NMR spectrum for fraction TBHeFRII that was elucidated as stigmasterol. Figure S4. The FTIR spectrum for fraction TBHeFRII that was elucidated as stigmasterol. Table S1. The 1H-NMR and 13C-NMR spectral analysis of TBHeFRII in CDCl3 at 500 MHz when compared with the literature [23, 24]. Table S2. FTIR spectral data showing the functional groups detected in TBHeFR5II when compared with the literature [25]. [file 6547734.f1.zip › 6547734.f1/TABLE S1.docx]

Table S1. The ^1^H-NMR and ^13^C-NMR spectral analysis of TBHeFRII in CDCl_3_ at 500 MHz when compared with the literature (23, 24).

| **Position** | **^1^H-NMR shift / ppm** (Isolated Compound) | **^1^H-NMR shift / ppm** (Lit. value) | **^13^C-NMR shift / ppm** (Isolated Compound) | **^13^C-NMR shift / ppm** (Lit. value) |
| --- | --- | --- | --- | --- |
| 1 | 1.35, m | 1.08, m | 39.69 | 39.66 |
| 2 | 1.83, m | 1.83, m | 31.66 | 31.61 |
| 3 | 3.55, m; 7.25, s | 3.51, m | 71.83 | 71.72 |
| 4 | 1.65, m | 2.30, m | 42.29 | 42.25 |
| 5 | 5.34, m | - | 140.76 | 140.67 |
| 6 | 5.45, d | 5.35, m | 121.75 | 121.62 |
| 7 | 1.98, m | 1.97, m | 31.90 | 31.87 |
| 8 | 1.95, d | 1.46, m | 37.26 | 37.24 |
| 9 | 0.95, m | 0.94, m | 50.13 | 50.12 |
| 10 | 1.05, s | 1.00, s | 40.52 | 40.50 |
| 11 | 2.04, d | 1.50, m | 21.23 | 21.22 |
| 12 | 2.00, m | 2.00, m | 45.83 | 42.18 |
| 13 | 0.65, s | 0.67, s | - | 42.20 |
| 14 | 1.00, m | 1.00, m | - | 57.10 |
| 15 | 1.60, m | 1.56, m | 24.32 | 24.35 |
| 16 | 2.25, m | 1.72, m | 28.94 | 28.92 |
| 17 | 1.15, q | 1.15, q | 55.95 | 55.91 |
| 18 | 0.70, s | 0.70, s | 11.83 | 12.04 |
| 19 | 1.01, s | 1.01, s | 19.42 | 19.39 |
| 20 | 0.95, d | 0.91, d | 56.77 | 56.83 |
| 21 | 1.03, d | 1.03, d | 21.09 | 21.09 |
| 22 | 5.05, d | 5.03, dd | 138.34 | 138.25 |
| 23 | 5.20, d | 5.13, dd | 129.27 | 129.19 |
| 24 | 1.55, m | 1.54, m | 51.25 | 51.21 |
| 25 | 0.83, m | 0.81, d | 18.76 | 18.98 |
| 26 | 0.85, d | 0.85, d | 20.64 | 21.06 |
| 27 | 0.80, d | 0.80, d | 19.42 | 19.26 |
| 28 | 1.43, m | 1.43, m | 25.43 | 25.40 |
| 29 | 0.81, t | 0.81, t | - | 12.20 |

Key: s- singlet d-doublet dd-double doublet m-multiplet t-triplet
