# Supplementary material for: Validating the Hypoglycaemic and Hypotensive Roles of Salvia serotina (Chicken Weed) in Normal Healthy Sprague–Dawley Rats
Source: ScientificWorldJournal. 2022 Jun 29;2022:6547734. doi: 10.1155/2022/6547734 (PMC9259359; doi:10.1155/2022/6547734)
Supplement: Supplementary Materials — Figure S1. The gas chromatogram of TBHeFR3 showed two bioactive compounds of interest, namely 3,7,11-trimethyl-1,6,10-dodecatrien-3-ol (1) commonly called Nerolidol and 3,7,11-trimethyl-2,6,10-dodecatrien-1-ol (2) commonly called Farnesol. Figure S2. The 1H-NMR spectrum for fraction TBHeFRII that was elucidated as stigmasterol. Figure S3. The 13C-NMR spectrum for fraction TBHeFRII that was elucidated as stigmasterol. Figure S4. The FTIR spectrum for fraction TBHeFRII that was elucidated as stigmasterol. Table S1. The 1H-NMR and 13C-NMR spectral analysis of TBHeFRII in CDCl3 at 500 MHz when compared with the literature [23, 24]. Table S2. FTIR spectral data showing the functional groups detected in TBHeFR5II when compared with the literature [25]. [file 6547734.f1.zip › 6547734.f1/TABLE S2.docx]

Table S2. FTIR spectral data showing functional groups detected in TBHeFR5II when compared with the literature (25).

| **Carbon Atom** | **Frequency / cm^-1^**  (Isolated Compound) | **Frequency / cm^-1^**  (Lit. value) | **Functional Group Present** |
| --- | --- | --- | --- |
| 1 | 639.95 | 667.0 | Cyclic C-H bending |
| 2 | 735.95 | 777 | Cyclic C-H bending |
| 3 | 836.98 | 881.6 | Cyclic C-H bending |
| 5,6 | 960.88 | 1036 | sp^2^ C-H bend of C=C |
| 3 | 1055.96 | 1089 | C-O stretch |
| 10 | 1373.24 | 1381.6 | CH(CH_3_)_2_ C-H stretch |
| 19,21,27 | 1457.35 | 1457 | C-H bending of CH_3_ |
| 19,21,27 | 1538.00 | 1504.47 | C-H bending of CH_3_ |
| 5,6 | 1701.79 | 1667 | Cyclic C=C absorption |
| 19,21,27 | 2859.46 | 2866 | sp^3^ C-H stretch |
| 19,21,27 | 2937.48 | 2934 | sp^3^ C-H stretch |
| 22,29 | 3103.64 | - | Sp^2^ C-H stretch of C=C |
| 5,6 | 3293.16 | - | Cyclic C=C stretch |
| 3 | 3460.20 | 3320 | O-H stretch |
|  | 3536.94 | - | O-H bond vibration |
