# Supplementary figures and images for: Validating the Hypoglycaemic and Hypotensive Roles of Salvia serotina (Chicken Weed) in Normal Healthy Sprague–Dawley Rats
Source: ScientificWorldJournal. 2022 Jun 29;2022:6547734. doi: 10.1155/2022/6547734 (PMC9259359; doi:10.1155/2022/6547734)

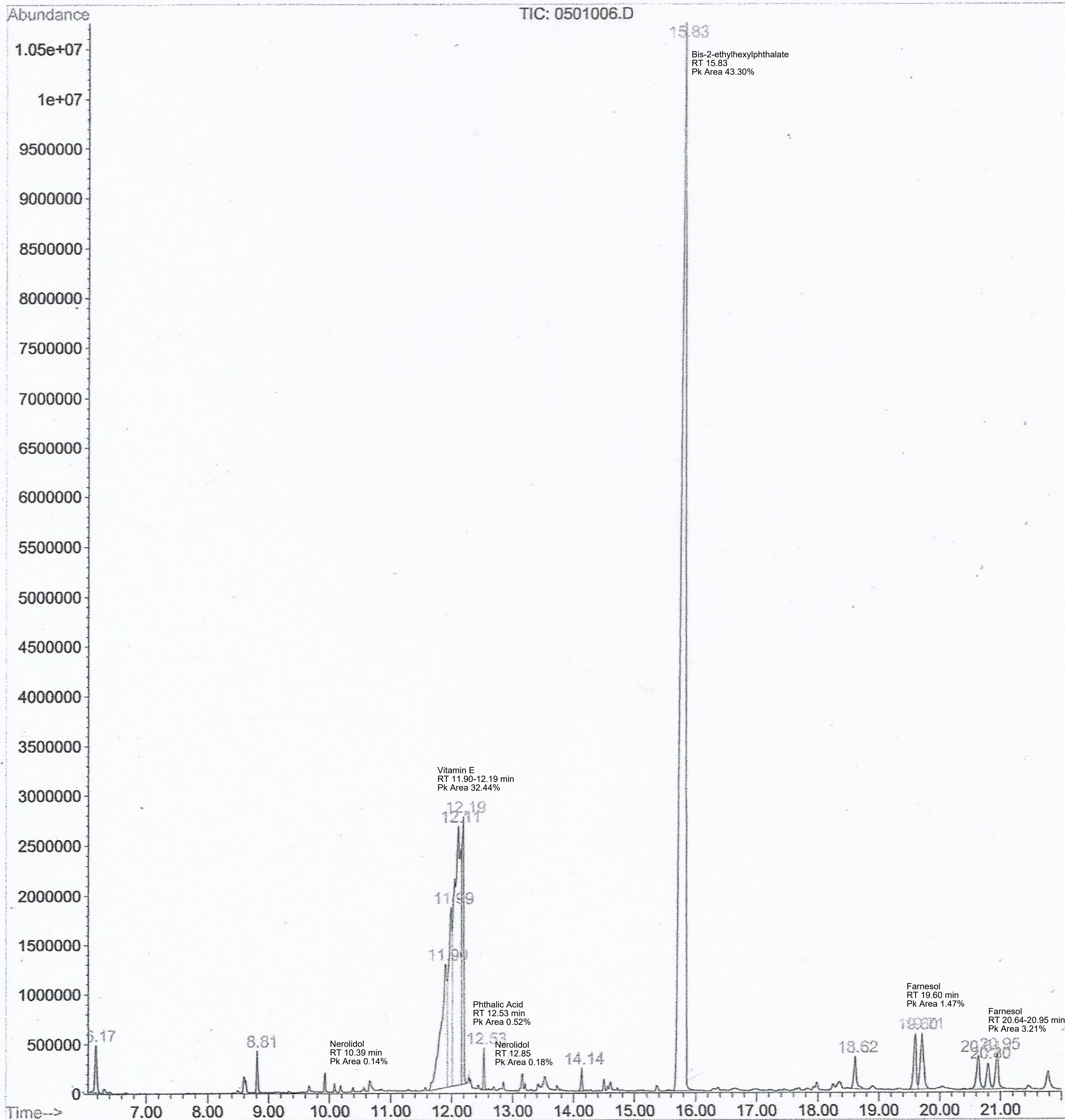

Supplement: Supplementary Materials — Figure S1. The gas chromatogram of TBHeFR3 showed two bioactive compounds of interest, namely 3,7,11-trimethyl-1,6,10-dodecatrien-3-ol (1) commonly called Nerolidol and 3,7,11-trimethyl-2,6,10-dodecatrien-1-ol (2) commonly called Farnesol. Figure S2. The 1H-NMR spectrum for fraction TBHeFRII that was elucidated as stigmasterol. Figure S3. The 13C-NMR spectrum for fraction TBHeFRII that was elucidated as stigmasterol. Figure S4. The FTIR spectrum for fraction TBHeFRII that was elucidated as stigmasterol. Table S1. The 1H-NMR and 13C-NMR spectral analysis of TBHeFRII in CDCl3 at 500 MHz when compared with the literature [23, 24]. Table S2. FTIR spectral data showing the functional groups detected in TBHeFR5II when compared with the literature [25]. [file 6547734.f1.zip › 6547734.f1/FIGURE S1.pdf]

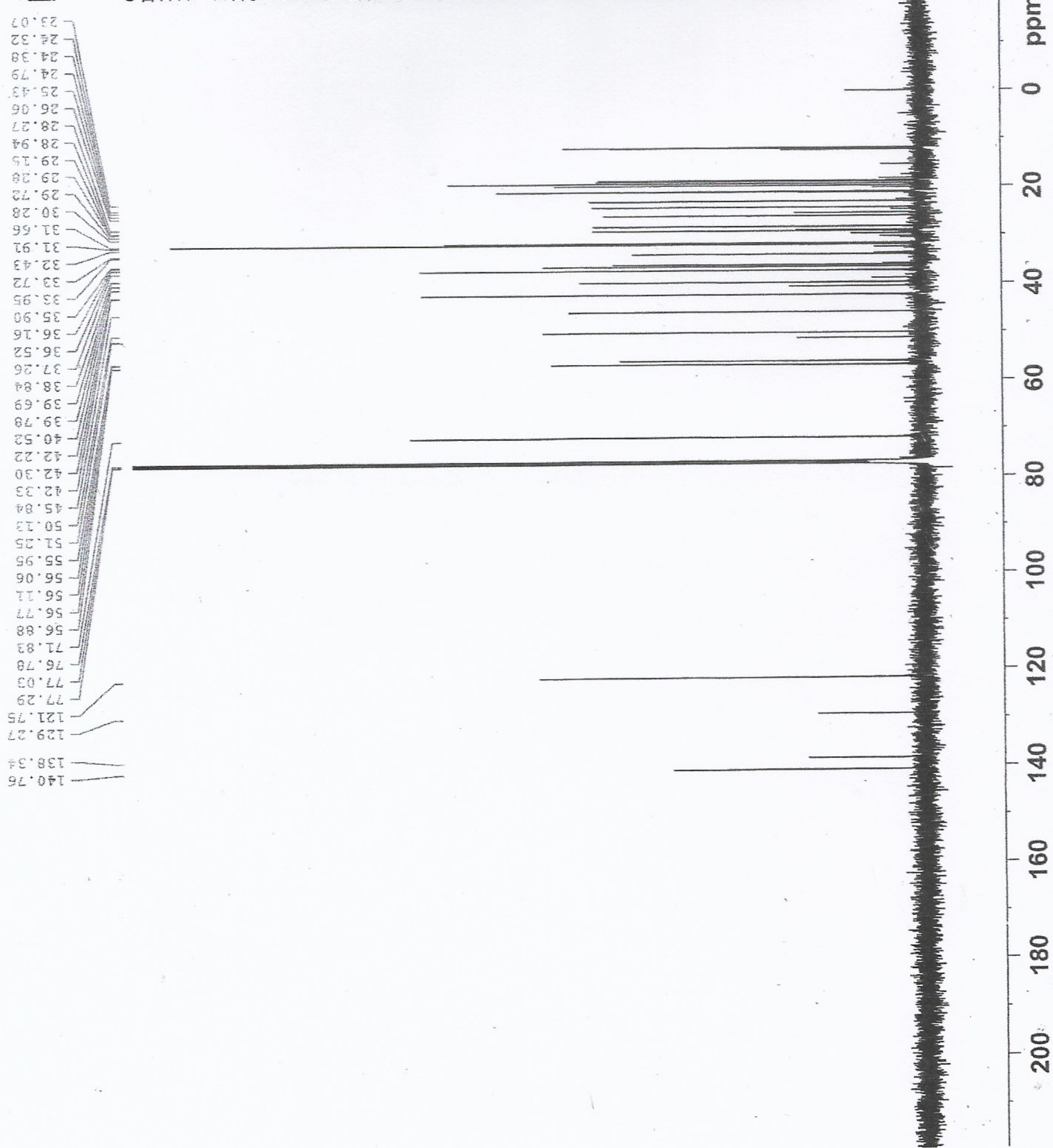

Supplement: Supplementary Materials — Figure S1. The gas chromatogram of TBHeFR3 showed two bioactive compounds of interest, namely 3,7,11-trimethyl-1,6,10-dodecatrien-3-ol (1) commonly called Nerolidol and 3,7,11-trimethyl-2,6,10-dodecatrien-1-ol (2) commonly called Farnesol. Figure S2. The 1H-NMR spectrum for fraction TBHeFRII that was elucidated as stigmasterol. Figure S3. The 13C-NMR spectrum for fraction TBHeFRII that was elucidated as stigmasterol. Figure S4. The FTIR spectrum for fraction TBHeFRII that was elucidated as stigmasterol. Table S1. The 1H-NMR and 13C-NMR spectral analysis of TBHeFRII in CDCl3 at 500 MHz when compared with the literature [23, 24]. Table S2. FTIR spectral data showing the functional groups detected in TBHeFR5II when compared with the literature [25]. [file 6547734.f1.zip › 6547734.f1/FIGURE S3.pdf]

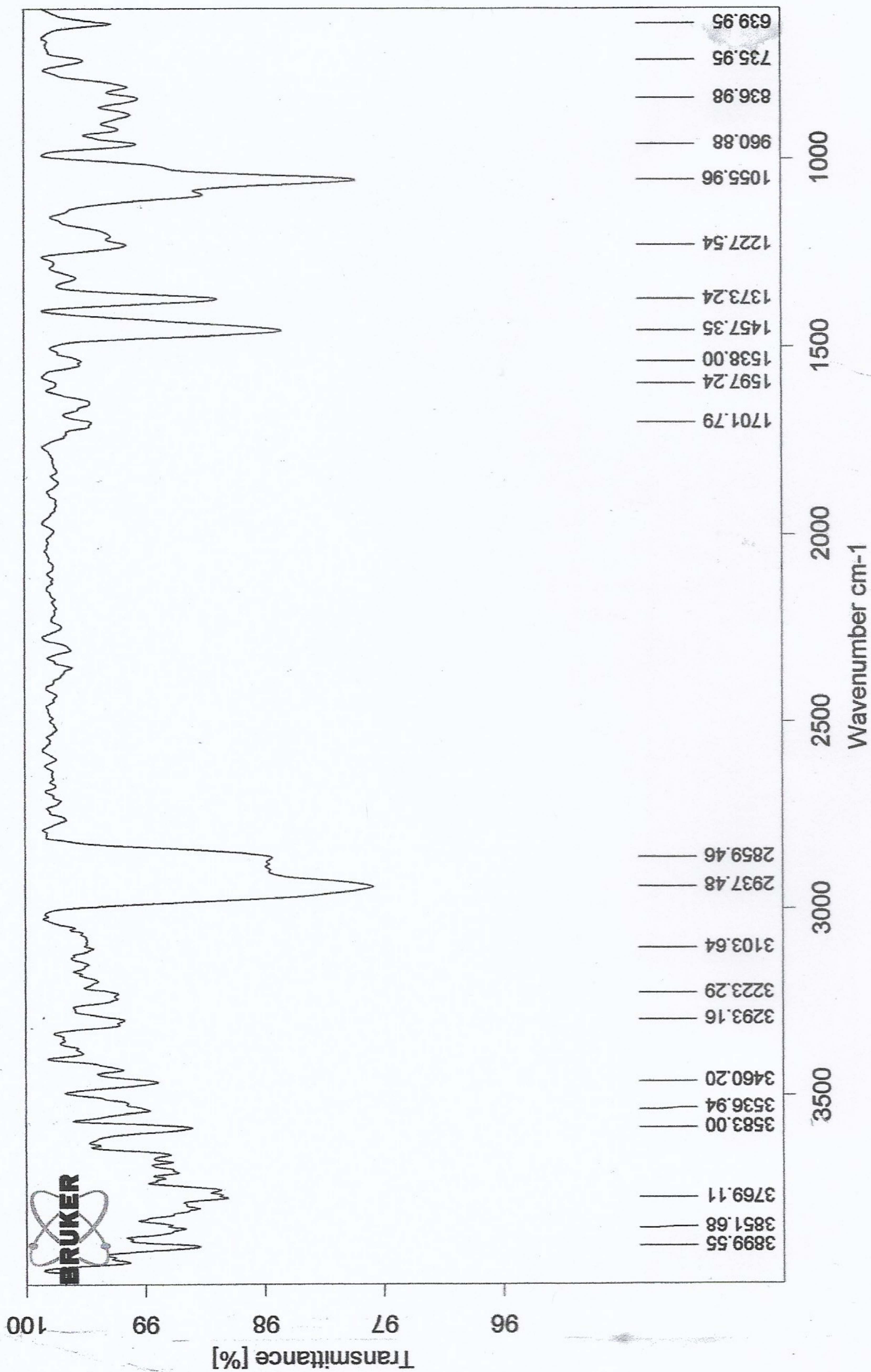

Supplement: Supplementary Materials — Figure S1. The gas chromatogram of TBHeFR3 showed two bioactive compounds of interest, namely 3,7,11-trimethyl-1,6,10-dodecatrien-3-ol (1) commonly called Nerolidol and 3,7,11-trimethyl-2,6,10-dodecatrien-1-ol (2) commonly called Farnesol. Figure S2. The 1H-NMR spectrum for fraction TBHeFRII that was elucidated as stigmasterol. Figure S3. The 13C-NMR spectrum for fraction TBHeFRII that was elucidated as stigmasterol. Figure S4. The FTIR spectrum for fraction TBHeFRII that was elucidated as stigmasterol. Table S1. The 1H-NMR and 13C-NMR spectral analysis of TBHeFRII in CDCl3 at 500 MHz when compared with the literature [23, 24]. Table S2. FTIR spectral data showing the functional groups detected in TBHeFR5II when compared with the literature [25]. [file 6547734.f1.zip › 6547734.f1/FIGURE S4.pdf]
